# A Self-Decoupling Technique to Realize Dense Packing of Antenna Elements in MIMO Arrays for Wideband Sub-6 GHz Communication Systems

**DOI:** 10.3390/s23020654

**Published:** 2023-01-06

**Authors:** Shahid Khan, Safdar Nawaz Khan Marwat, Muhammad Amir Khan, Salman Ahmed, Neelam Gohar, Mohammad Ehsanul Alim, Abeer D. Algarni, Hela Elmannai

**Affiliations:** 1 Department of Electrical and Computer Engineering, COMSATS University Islamabad, Abbottabad Campus, Abbottabad 22060, Pakistan; 2 Department of Computer Systems Engineering, Faculty of Electrical and Computer Engineering, University of Engineering and Technology Peshawar, Peshawar 25120, Pakistan; 3 Department of Computer Science, COMSATS University, Islamabad-Abbottabad Campus, Abbottabad 22060, Pakistan; 4 Department of Computer Science, Shaheed Benazir Bhutto Women University, Peshawar 25000, Pakistan; 5 Department of Electrical & Computer Engineering, University of Delaware, Newark, DE 19716, USA; 6 Department of Information Technology, College of Computer and Information Sciences, Princess Nourah bint Abdulrahman University, P.O. Box 84428, Riyadh 11671, Saudi Arabia

**Keywords:** antenna array, circular patch antenna, multiple-input multiple-output (MIMO), mutual coupling, envelope correlation coefficient (ECC), sub-6 GHz wireless communication systems

## Abstract

A self-decoupled technique is described that enables the radiating elements in the antenna array to be densely packed for multiple-input multiple-output (MIMO) wireless communications systems. High isolation between the adjacent antenna elements is obtained by fixing the radiating elements in an orthogonal configuration with respects to each other. Current from the adjacent ports cancels their impact which results in low mutual coupling. The additional benefit of this configuration is realizing a densely packed array. The ground plane of each radiating element on the array board itself are isolated to mitigate surface wave propagations to suppress mutual coupling between the antenna elements. The radiating elements are based on a modified edge-fed circular patch antenna that includes a curved slot line and open-circuited stub to widen the array’s impedance bandwidth with no impact on the antenna’s footprint size. The proposed technique was verified with the design of an antenna array of matrix size 4 × 4 centered at 3.5 GHz. The array had a measured impedance bandwidth of 4 GHz from 1.5 GHz to 5.5 GHz, which corresponds to a fractional bandwidth of 114%, peak gain of 3 dBi and radiation efficiency of 84%. Its average diversity gain and envelope correlation coefficient (ECC) over its operating band are 9.6 dB and <0.016, respectively. The minimum isolation achieved between the radiating elements is better than 15 dB. The dimensions of the array are 0.4 × 0.4 × 0.039λ_g^3. The proposed array has characteristics suitable for sub-6 GHz wireless communication systems

## 1. Introduction

In 5G wireless communication systems, MIMO antenna arrays play a key role in enabling better coverage and higher data rate transmission. Antenna arrays enable beam forming and massive multiple-input multiple-output (MIMO), which are essential for 5G and subsequent generations of wireless communication systems. MIMO is based on the concept of multipath propagation, which states that a signal from a single source can reach end users via multiple paths. MIMO uses multiple low data rate sub-signals to transmit a single signal over a single frequency channel using spatially separated antennas. They arrive at the receiver via different paths due to multipath propagation. These signals are divided into parallel streams by the receiver, which are then processed to recover the original signal. With the MIMO technology the channel capacity can be significantly improved without the need for additional bandwidth and power consumption.

Massive MIMO necessitates a large number of antennas that are sufficiently spaced apart to prevent crosstalk between channels. It is challenging to separate antenna elements in a very large array for real-time applications without experiencing a significant increase in array size. Therefore, the challenge facing antenna designers is to squeeze many antenna elements in a small space however, the limiting factor is strong mutual coupling between the antennas that can severely deteriorate the far field patterns of the radiating elements in the MIMO antennas. As a result, suppressing mutual coupling in sub-6 GHz base-station MIMO antenna arrays is becoming more and more important. In order to reduce the mutual coupling, a variety of techniques have been investigated, such as suppressing surface or space waves between antenna elements using defective ground structures (DGS) [1,2], employing electromagnetic bandgap (EBG) structures [3,4,5], metamaterials [6,7,8], or to counteract the inherent coupling with introduction of additional coupling paths among the radiating elements. This is done using neutralization lines [9,10], parasitic elements [11,12], or decoupling networks [13,14]. These techniques are based on extra decoupling structures, which considerably increase the design complexity and footprint of the MIMO array and even adversely affect its far field radiation characteristics. More recently, self-decoupling antenna arrays have been investigated [15,16,17,18]. Instead of using external decoupling structures, this technique reduces mutual coupling by utilizing the intrinsic characteristics of the antenna. For example, the inset-fed patch antenna described in [19] cleverly uses the coupling field from the feeding structure to counteract the counterpart from the radiating patch, the inverted-F antenna in [20] uses the cancellation effect between the common and differential modes, while the rectangular patch antenna in [21] makes use of the combination of TM1,0 and TM0,2 modes to decouple. Amazingly, it is demonstrated that these designs can reduce mutual coupling without the use of any additional decoupling structures. However, it should be noted that all the above techniques apply to linearly polarized arrays. CP antennas are more useful for real-time applications than linearly polarized ones because they can reduce polarization mismatch, suppress multipath interference, and overcome the Faraday effect [22,23]. The literature on coupling reduction in circularly polarized antenna arrays is very limited, and they all make use of extra decoupling methods, such as the EBG structure [24], DGS [25], and others, leading to complex designs.

For wideband sub-6GHz MIMO communication systems like 5G and 6G, a self-decoupling technique is proposed in this paper that enables the realization of a compact antenna array. This is accomplished by (i) arranging the radiating elements orthogonally with respect to one another, (ii) isolating the ground plane of each radiating element on the array board itself to mitigate surface wave propagation and thus suppress unwanted mutual coupling between the radiating elements, and (iii) embedding a curved slot line inside the radiating patch and creating an open-circuited stub to increase the antenna’s aperture area. The normal technique to reduce surface waves is by defecting the ground plane with electromagnetic bandgap structures and inserting a decoupling structure between the radiating elements, which compromises the footprint size of the array. In the proposed technique, the orthogonal arrangement of radiating elements results in circularly polarized transmission. The innovation presented here in this work is the compact size which is obtained due to multiple slits and slots and high inter elements isolation due to orthogonal arrangement of the radiating elements. The effectiveness of the proposed technique is demonstrated with a prototype antenna array of matrix size 2 × 2 designed at 3.5 GHz on a standard FR-4 substrate.

The remaining paper is organized as follows: Section 2 describes the proposed antenna design and parametric study, which were carried out to determine the key structural parameters influencing its performance. Section 3 goes over the specifics of the MIMO antenna array configuration and its performance. Section 3 also gives details of the proposed MIMO array’s time domain analysis and comparison of the proposed antenna design to other works, and Section 4 concludes the work

## 2. Design of the Unit Cell

**Figure 1:**
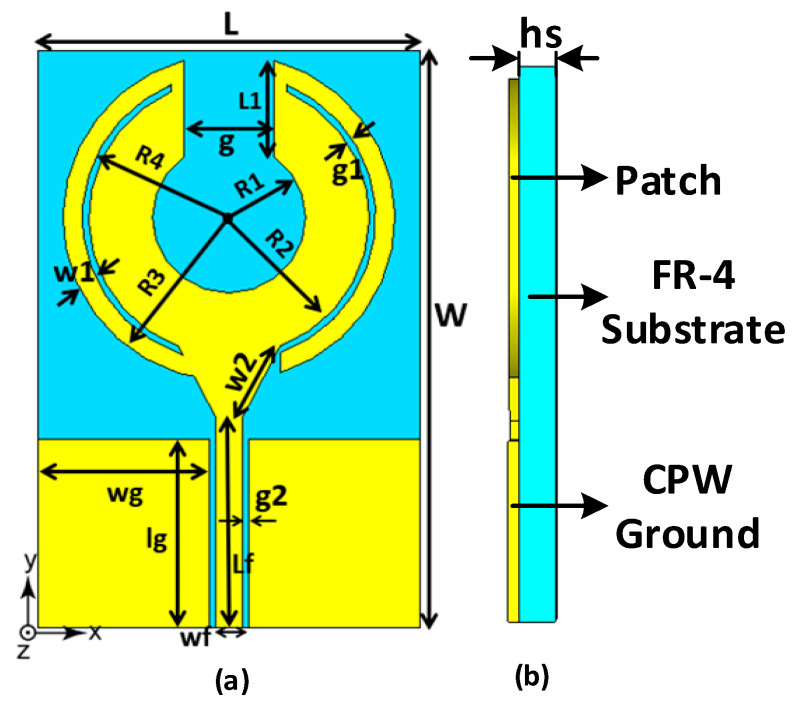
(**a**) Shows the diagram of unit cell of the MIMO antenna, and (**b**) shows the antenna’s side view.

In the proposed orthogonal MIMO antenna array, the schematic diagram of the unit cell employed is shown in Figure 1. The unit cell is based on a regular circular patch antenna, which has been modified with the inclusion of a curved slot line, a curved open-circuited stub, and by cutting out a shape analogous to a rounded-bottom flask. The slot line and open-circuited stub essentially enhance the antenna’s aperture area and reflection coefficient, i.e., impedance match. The unit cell resembles a thick split-ring structure that is edge-fed through a coplanar waveguide. The design of the circular patch antenna follows a standard procedure. The radius “a” can be calculated using [12].

a = F⁄√(1 + 2h/(πε_r F) [ln⁡(Fπ/2h) + 1.7726])(1)

where F = (8.791 × 〖10〗^9)⁄(f_r √(ε_r))

“ε_r” is the relative permittivity of the substrate, “fr” is the resonant frequency, and “h” is the thickness of the substrate. The array design was centered at 3.5 GHz and fabricated on an FR-4 substrate with 1.6 mm thickness 4.4 relative permittivity and 0.002 loss tangent. The design was simulated and its performance optimized for operation between 1.5 to 5.5 GHz using CST Microwave Studio, which is a 3D full wave electromagnetic solver. CST Microwave Studio is based on FIT (Finite Integration Technique) and TLM (Transition Line Matrix) in the time domain and FEM (Finite Element Method) in the frequency domain. The actual and guided wavelength dimensions of the optimized design are in Table 1. 

### 2.1. Design Steps

**Figure 2:**
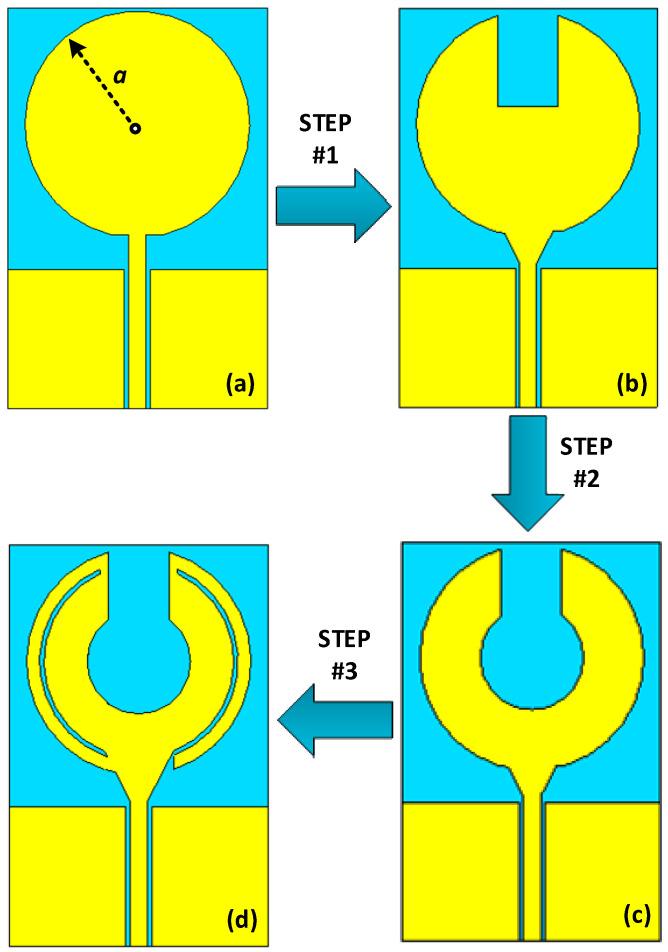
Design procedure of the proposed MIMO antenna unit cell, (**a**) Standard circular antenna with a coplanar waveguide feedline, (**b**) circular patch antenna with cutout rectangular section and widened edge feed point, (**c**) circular patch in step #1 with circular section cutout, and (**d**) Circular patch in step #2 with embedded curved slot lines on both sides of the patch however the slot line on the right-hand side is cut to create a curved open-circuited stub.

**Figure 3:**
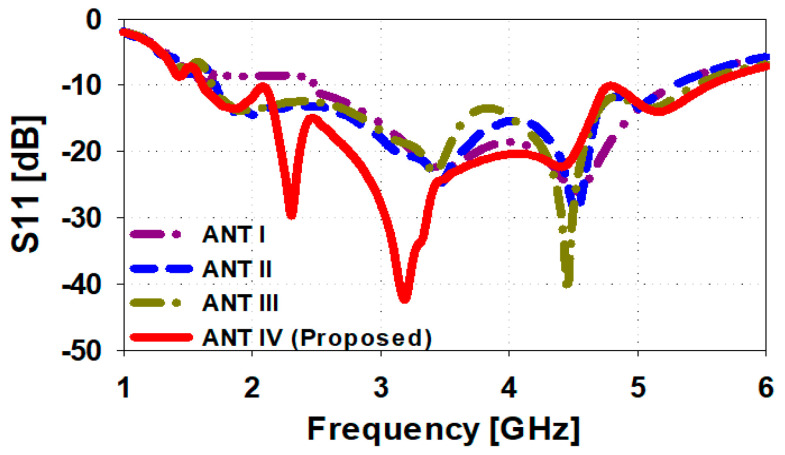
Reflection-coefficient of the circular patch antenna corresponding to the four steps in Figure 2 to realize the proposed unit cell antenna.

Figure 2 illustrates the steps taken to design the proposed unit cell antenna. Figure 3 give details of the reflection-coefficients of the corresponding design steps. The design begins with a traditional edge-fed microstrip patch antenna of a circular configuration that is excited through a coplanar waveguide (CPW) feedline. The impedance bandwidth of the circular patch antenna is 2.7 GHz from 2.5 GHz to 5.2 GHz for S11 ≤ −10 dB. In step #1, a rectangular section is cutout from upper part of the radiating antenna and the edge-fed section of the feedline is widened to improve the antenna’s impedance match. Figure 3 shows that the reflection coefficient of the antenna in step #1 is increased to 3.52 GHz from 1.58 GHz to 5.1 GHz. In step #2, a circular section is cutout at the center of the patch to create a shape that resembles a rounded-bottom flask shape. Figure 3 shows by doing this the impedance bandwidth is increased to 3.82 GHz from 1.58 GHz to 5.4 GHz. In the final step #3, the patch antenna is embedded with curved slot lines on the both sides of the patch however, the slot line on the right-hand side is directly merged with the feedline thus creating a curved open-circuited stub. By doing this, the impedance bandwidth for -10db reference value is extended to 4 GHz (from 1.5 GHz to 5.5 GHz). It is evident from Figure 3 that by implementing step #3 the matching performance has significantly improved. In Comparison to the traditional circular patch antennas the proposed design provides 48.1% increase in the operating bandwidth and significantly improved reflection-coefficient. This performance enhancement is achieved without increasing the antenna’s footprint size. 

### 2.2. Parametric Study of the Design 

**Figure 4:**
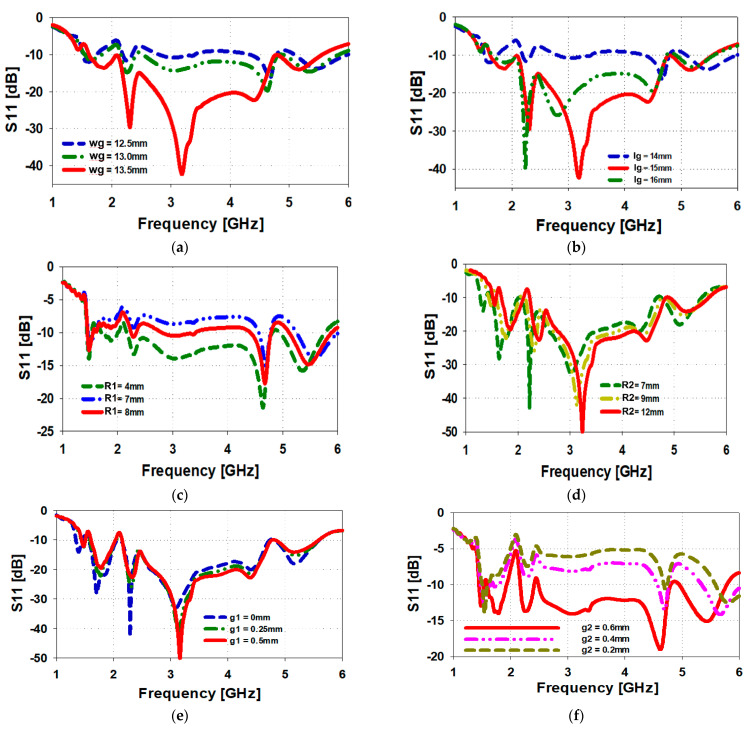
(**a**) Effect of variation in “wg” on S11, (**b**) effect of variation in “Ig” on S11, (**c**) effect of variation in “R1” on S11, (**d**) effect of variation in “R2” on S11, (**e**) effect of variation in “g1” on S11, and (**f**) effect of variation in “g2” on S11.

The proposed antenna’s performance depends on number of parameters. Figure 4 show how the reflection-coefficient response of the antenna is affected by the ground plane width “wg”, ground plane length “Lg”, inner slot radius “R1”, outer patch radius “R2”, slot width “g1”, and the gap between feedline and ground “g2”. Figure 4a shows by increasing the ground plane width “wg” from 12.5 mm to 13.5 mm the impedance match is significantly improved. However, any further increase in the ground plane width undermines the impedance bandwidth of the antenna. Increasing the ground plane length “Lg” from 14 mm to 15 mm, as shown in Figure 4b, also significantly increases the antenna’s reflection coefficient, however any further increase in "Lg” tends to deteriorate the antenna’s matching performance. The optimum ground plane length for the proposed antenna design is 15 mm. Figure 4c shows the inner slot radius “R1” significantly affects the impedance bandwidth. With R1 of 7 mm, the impedance match on average is −7 dB, and when R1 is 8 mm the average match improves to approximately −10 dB, however, R1 is 4 mm, it provides the best match with an average match of −12.5 dB. Figure 4d shows the outer patch radius “R2”. A radius of 12 mm provides a better match than 7 mm but this is for frequencies above 3.3 GHz. The converse applies to frequencies below 3.3 GHz. The outer slot gap “g1” has a marginal effect on the reflection-coefficient response of the antenna, as shown in Figure 4e. However, the gap between the feedline and ground “g2” has a dramatic effect on the antenna’s wideband matching performance, as shown in Figure 4f. It is evident from this figure that the tighter the gap the better the match. A gap of 0.2 mm provides the best impedance match. 

## 3. MIMO Arrangement of the Design

**Figure 5:**
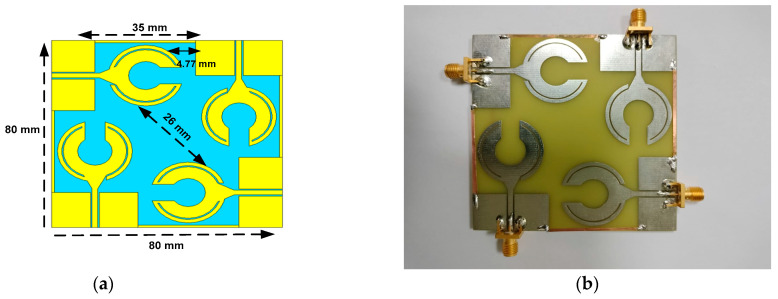
MIMO antenna shown in (**a**), fabricated 2 × 2 antenna array prototype shown in (**b**).

The proposed MIMO design dimension and fabricated antenna array are shown in Figure 5a,b, respectively. It shows how the proposed patch antennas are configured on the board. A 35 mm gap exists among ports of the radiating elements which are connected by 1 mm thick copper lines. The diagonal distance among the radiating elements is 26 mm. Self-decoupling is achieved by arranging the antennas orthogonally. Unlike other techniques, the proposed technique does not require the embedding of decoupling structures between the antennas or defecting the ground plane with electromagnetic bandgap structures. This technique significantly reduces the mutual coupling between the adjacent antennas that permits antennas in the array to be squeezed tightly thus enabling the reduction in the form factor of the MIMO system. On the array board itself, the ground plane of each radiating element is connected to others by a 1 mm thin copper line which helps to mitigate surface wave propagations and thereby suppress unwanted mutual coupling between the radiating elements. The normal technique to reduce surface waves is to insert EBG structures between the radiating elements. This is important to dissipate the build of high potential at the excitation ports resulting from induced voltage standing waves that can potentially break down the ends of the cable, damaging connectors, and antennas. The size of the 4 × 4 array is 80 × 80 × 1.6 mm^3^.

### 3.1. Performance of Antenna Array

**Figure 6:**
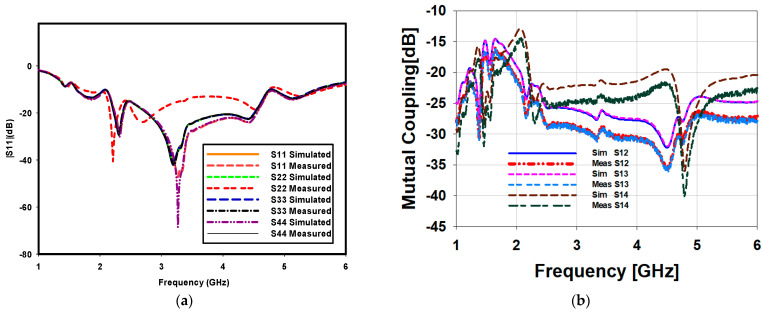
(**a**) Simulated and measured reflection coefficients, and (**b**) simulated and measured mutual coupling between the array ports.

The S-parameter response of the proposed antenna array was validated using a vector network analyzer (VNA), which was calibrated to remove systematic errors from the instrument hardware. During the collection of the measured data, two ports of the proposed design were connected with coaxial probes and other two ports were terminated with 50 Ω terminators. Figure 6a shows the measured impedance bandwidth is 4 GHz from 1.5 GHz to 5.5 GHz for S_11_ ≤ −10 dB reference value. There is excellent agreement between the simulated and measured reflection-coefficients response of the array. The marginal discrepancies in the impedance matching of the four ports are attributed due to the fabrication tolerances, surrounding noise and non-uniform soldering. The impedance bandwidth at all ports have been noted. The impedance matching of the four ports are different however, the impedance bandwidth has been retained. At port 2 there is slight decrease in the measured bandwidth. The simulated and measured mutual coupling between the various ports of the array is shown in Figure 6b. It is noticed that around 1.5 GHz, the isolation is around 15 dB, while for large part of the operating bandwidth the isolation is around 20 dB. Thus, it can be concluded that at low frequencies, there is some current flow between the ports while at higher frequencies most of the current is directed toward the antenna radiators. It is also noticed that the simulated and measured mutual coupling are also in close agreement. It is important to note that this much isolation is due to the orthogonal arrangement of the antenna elements. Adding any additional decoupling technique will further enhance the isolation among the antenna elements which can be a future research activity. 

### 3.2. Surface Current and Electric Fields Distribution

**Figure 7:**
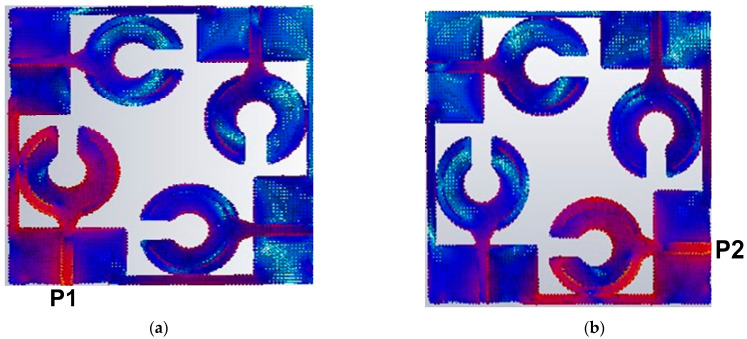
Surface current density distribution at 3.5 GHz over the proposed antenna array, (**a**) excitation at port 1 and (**b**) excitation at port 2.

**Figure 8:**
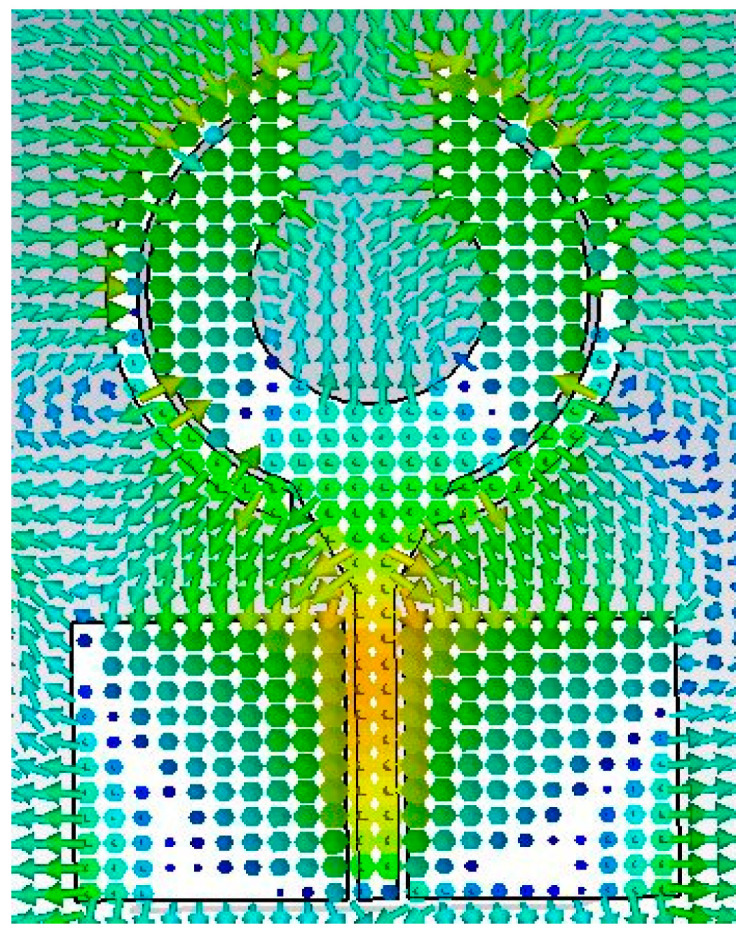
Electric field distribution of the proposed patch antenna at 3.5 GHz.

The current distribution on the surface of the radiator explains well the inter port isolation while the electric field distribution identify the operating mode on the patch which is shown in Figure 7a,b. Figure 7a shows that when port #1 is excited the current is heavily concentrated on the port and peripheries of the patch antenna next to the curved slot line and the open-circuited stub. It is observed that some current is induced in other antennas in the array but marginally more in the antenna in port #1. Moreover, the low current activity is noticed in nearby elements due to port 1 which shows good isolation between the antenna elements. Figure 7b shows similar current distribution is obtained when the antenna in port #2 is excited. Figure 7 also shows that thin lines of 1 mm that connect the ground planes of all the radiating elements cause a minute amount of current flow between port 1 and 2. As a result, there is a slight reduction in the isolation at low frequencies. Figure 8 shows the electric field distribution at 3.5 GHz. The electric field at port 1 is noticed by terminating the other ports. The electric field activity is more on the top and bottom of the patch, thus these parts are contributing more to the radiation. Furthermore, the electric field distribution on the antenna demonstrates the excitation of higher order transverse.

### 3.3. Far-Field Radiation Patterns

**Figure 9:**
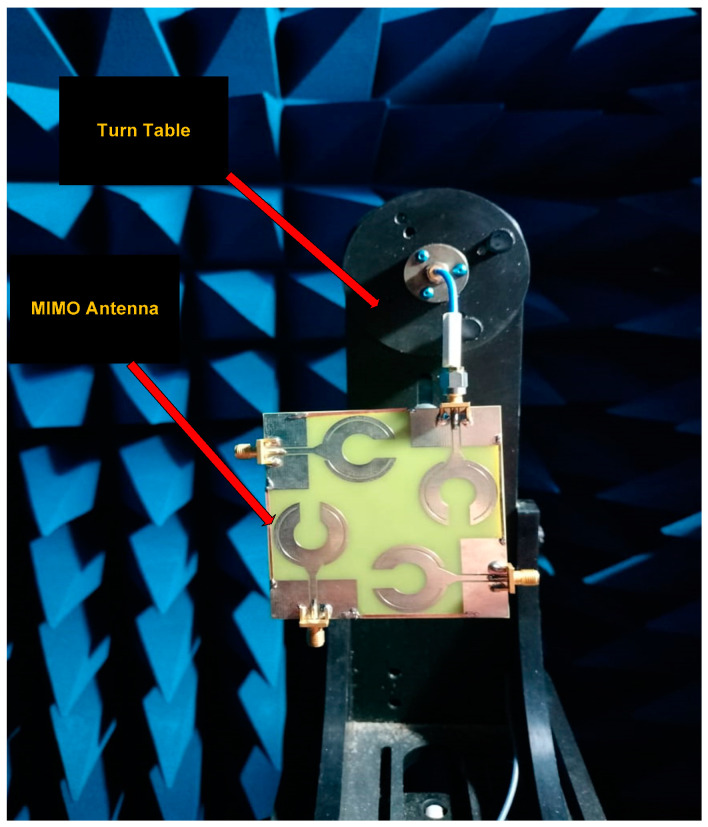
Radiation pattern measurement setup.

**Figure 10:**
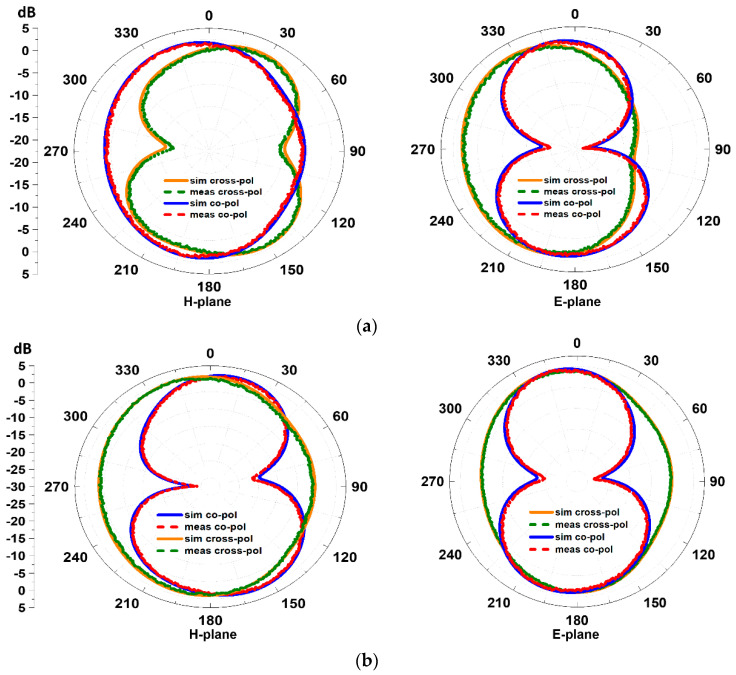
Shows the simulated and measured far-field H and E-plane patterns (**a**) at 2.4 and (**b**) at 3.5 GHz respectively when excited at port #1.

The tests were carried out in an anechoic chamber to eliminate reflections and the reception of spurious external signal which is shown in Figure 9. The array was set up on a turntable, and radiation patterns in the azimuthal (H-plane) and elevation (E-plane) planes were measured. Figure 10a,b depicts the simulated and measured co-polarization and cross-polarization radiation patterns of an antenna in both planes (H and E-plane) at 2.4 and 3.5 GHz respectiely. The co-polarization radiation in the H-plane has an oval shape, while the cross-polarization radiation has a figure eight shape. The cross-polarization radiation resembles a tighter figure eight shape in the E-plane, while the co-polarization radiation has an oval shape with compression on the right side. The array’s antennas are arranged orthogonally to one another, which results in identical co- and cross-polarization in the broadside direction. The far field distribution for both the resonance frequencies in the E-plane are directed towards 0^0^ and have low side lodes. The remarkable correlation between the simulated and measured results is noticed.

**Figure 11:**
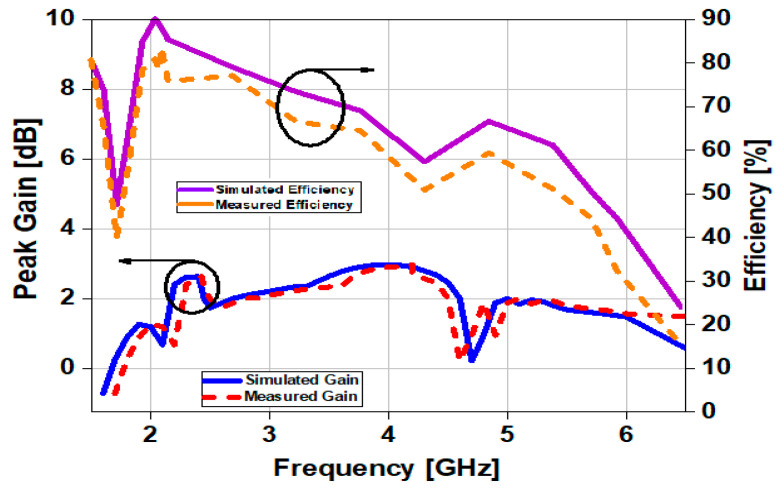
Shows Gain and efficiency of the proposed antenna have been measured and simulated.

The simulation and measured gain and radiation efficiency are given in Figure 11. At 2.4 GHz the measured the gain is 2.65 dBi, and at 3.5 GHz the gain is 2.5 dBi. At 4 GHz, 3 dBi of peak gain was observed. At 2.4 GHz, the radiation efficiency is measured to be 78%, while at 3.5 GHz, it is 67%. The measured peak efficiency at 2.2 GHz is 84%. Inaccurate fabrication and a flawed measurement setup are the main reasons for the discrepancy between the simulated and measured results.

### 3.4. Diversity Analysis

Diversity analysis is important to ascertain the quality and reliability of a wireless link. To compute the diversity gain it is necessary to determine the envelope correlation coefficient (*ECC*) of the antenna array. Low variation in radiation patterns and high isolation in array elements give low values of *ECC* and therefore result in high diversity gain (*DG*). *ECC* is determined using Equation (2) [26,27].

(2) $$\rho_{e}=\frac{|\int_{0}^{2\pi }\int_{0}^{\pi }(XPR.\;E_{\theta 1}.E_{\theta 2}^{*}.P_{\theta }+E_{\varphi 1}.\;E_{\varphi 2}^{*}.P_{\varphi })d\Omega {|}^{2}}{\int_{0}^{2\pi }\int_{0}^{\pi }(XPR.\;E_{\theta 1}.E_{\theta 1}^{*}.P_{\theta }+E_{\varphi 1}.\;E_{\varphi 2}^{*}.P_{\varphi })d\Omega \;\times \int_{0}^{2\pi }\int_{0}^{\pi }(XPR.\;E_{\theta 2}.E_{\theta 2}^{*}.P_{\theta }+E_{\varphi 2}.\;E_{\varphi 2}^{*}.P_{\varphi })d\Omega }$$

where *XPR* is the vertical-horizontal power discrimination ratio, $E_{\theta 1},E_{\theta 2}^{*}$ and $E_{\varphi 1}.\;E_{\varphi 2}^{*}$ are the electric field components in elevation and azimuth directions, respectively, and $P_{\theta }$ and $P_{\varphi }$ are the angular power in elevation and azimuth directions, respectively. *DG* is a function of *ECC* given by [27]

(3) $$DG=\sqrt{1-ECC^{2}}$$

**Figure 12:**
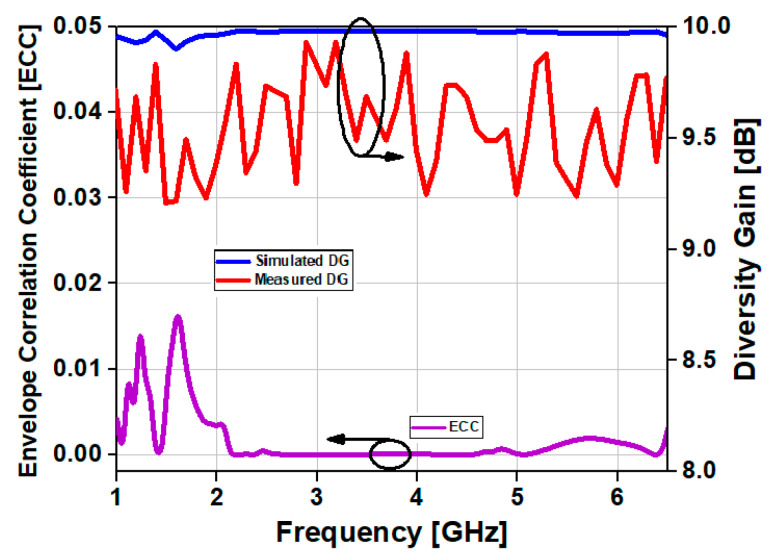
Shows the proposed design’s ECC and diversity gain.

A *GD* of 10 dB is the standard value, which shows that if this value is achieved, a decrease at the input power will not impact the regular transmission. Figure 12 shows the simulated and measured *DG* and *ECC*. The simulation was done using CST Microwave Studio The graph clearly shows that the simulated *ECC* of the proposed array is less than 0.016, which is excellent as compared to the 0.5 standard value. The average measured *DG* is 9.6 over the entire frequency span of interest which is close to the 10 dB standard value. 

**Figure 13:**
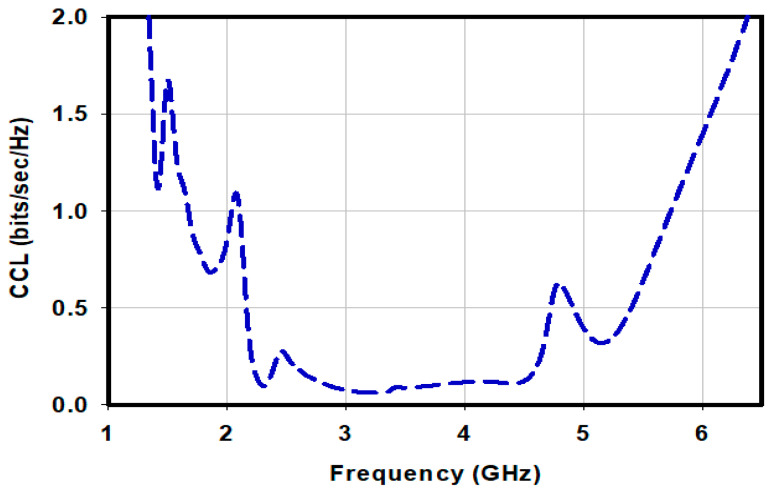
Shows the channel capacity loss of the proposed antenna.

An antenna array’s channel capacity loss (CCL) is used to determine the effectiveness of the transmission throughput. A good data transmission rate is indicated by smaller values of CCL. A CCL of 0.4 bits/s/Hz is accepted as good data transmission, however values greater than 0.4 bits/s/Hz indicate increasing inefficiency in the transmission. Figure 13 shows the CCL of the proposed array. At its lower operating band frequency of 1.5 GHz the value of CCL is 1.1 bits/s/Hz and at the upper end at 5.5 GHz the value of CCL is 0.7 bits/s/Hz. However, across 2.2 GHz to 4.54 GHz and between 4.9 GHz and 5.45 GHz the value of CCL is less than 0.4 bits/s/Hz. The average CCL across the array’s operating band is less than 0.4 bits/s/Hz. These excellent MIMO parameter values are a sign of the successful performance of the suggested design.

### 3.5. Time Domain Analysis of the Proposed Antenna Array

**Figure 14:**
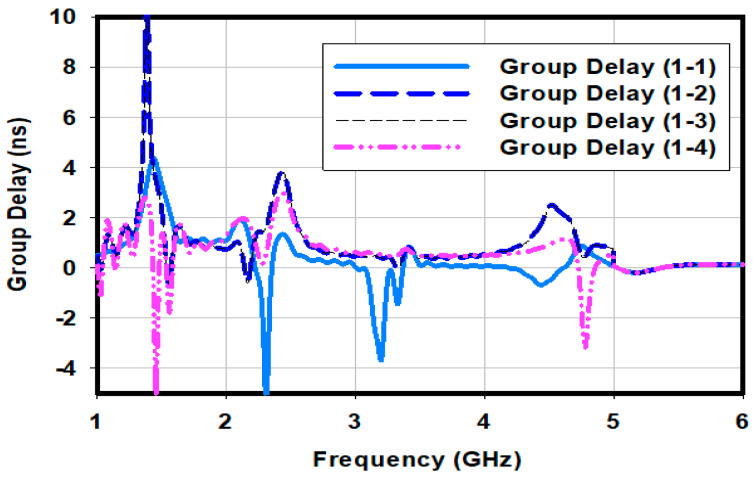
Group delay of proposed antenna array.

The group delay is a crucial time domain analysis parameter that describes how a signal’s various frequency components are delayed as the signal travels through a system. Figure 14 depicts the group delay of the proposed antenna array between port #1 and the other ports from 1 GHz to 6 GHz. In the operating band of the array between 1.5 GHz and 5.5 GHz the group delay is less than 4 ns. These values of the group delay over the entire operating band are considered good.

### 3.6. Comparison of the Proposed Antenna Array with Published Works

Comparisons of the characterizing parameters of the suggested antenna array with state-of-the-art published work is detailed in Table 2. The comparison is made based on the array’s footprint size, operating bandwidth (BW), peak gain, isolation, efficiency, *ECC* and decoupling technique employed. The proposed array operates over a very wide bandwidth and has a relatively small footprint, as shown in the table. Its radiation efficiency is much higher, and its *ECC* is the smallest with the exception of [28]. 

## 4. Conclusions

The effectiveness of the innovative technique is practically shown with a 4 × 4 design antenna array. The technique enables radiating elements in an antenna array to be more densely packed without compromising the array’s radiation characteristics and for achieving transmission over a wideband. This is important to reduce the form factor of MIMO systems. The isolation between the adjacent antennas is increased by arranging the radiating elements in an orthogonal configuration with respect to each other. The ground plane of each radiating element on the array board itself is isolated to suppress surface waves that otherwise would contribute towards unwanted mutual coupling. The measured results confirm the 4 × 4 antenna array operates across 1.5 GHz to 5.5 GHz with a peak gain of 3 dBi and peak efficiency of 84%. The array has an average diversity gain of 9.6 dB, ECC < 0.016, and isolation between the radiating elements of >15 dB over its operating band. These features make the proposed array suitable for 5G and 6G communication systems.

## Figures and Tables

**Table 1:** Design dimensions of the proposed unit cell antenna.

Antenna Parameters	Dimensions (mm)	Guided Wavelength (λg)	Antenna Parameters	Dimensions (mm)	Guided Wavelength (λg)
L	46	1.125	W	30	0.734
L1	7.65	0.187	w1	1.55	0.038
Ig	15	0.367	wg	13.5	0.330
Lf	16.65	0.407	wf	2	0.049
g	7.0	0.171	w2	6.49	0.159
g1	0.50	0.012	R1	6.0	0.147
g2	0.50	0.012	R2	11	0.269
R4	11.5	0.281	R3	13	0.318

**Table 2:** Comparison of proposed antenna array with previous works.

Ref. No.	Area $\left(\mathrm{mm})/(\lambda_{g}^{2}\right)$	Operating BW (GHz)	Peak Gain (dB)	Isolation (dB)	Peak Eff. (%)	ECC	Substrate Used	Target Application	Technique Used
[25]	150 × 75/ (3.52 × 1.76)	0.2	<5.1	16.5	82	0.01	FR-4	5G	L-shaped strip
[26]	150 × 75/ (3.52 × 1.76)	0.26	<1.6	>20	47	<0.3	FR-4	5G	M-shaped strip
[27]	24 × 22/ (0.45 × 0.45)	2.2	NA	15	NA	0.04	FR-4	5G	NA
[28]	145 × 75/ (3.4 × 1.76)	0.2	4.5	>15	73	<0.16	FR-4	5G	L-shaped deformation in the ground plane
[29]	154 × 154/ (5.32 × 5.32)	0.3	5	>40	-	-	FR-4	NM	Printed Yagi Uda
[30]	263 × 263/ (2.63 × 2.63)	1.1	7	18	65	<0.159	FR-4	GSM/UMTS/EDGE	Circular Quasi Yagi
**[This work]**	**80 × 80/** **(0.4 × 0.4)**	**4**	**3**	**>15**	**84**	**<0.016**	**FR-4**	**5G/Sub 6-GHz** **Wireless applications**	**Self-decoupling technique**

## Data Availability

No data were used to support this study. We have conducted simulations to evaluate the performance of the proposed protocol. However, any query about the research conducted in this paper is highly appreciated and can be asked by the author (Muhammad Amir Khan) upon request.
